# Glymphatic pathways in the gyrencephalic brain

**DOI:** 10.1177/0271678X21996175

**Published:** 2021-02-27

**Authors:** Nicholas Burdon Bèchet, Nagesh C Shanbhag, Iben Lundgaard

**Affiliations:** 1 Department of Experimental Medical Science, Lund University, Lund, Sweden; 2 Wallenberg Centre for Molecular Medicine, Lund University, Lund, Sweden

**Keywords:** Glymphatic system, pig model, cerebrospinal fluid, tissue clearing, light sheet microscopy

## Abstract

Identification of the perivascular compartment as the point of exchange between cerebrospinal fluid (CSF) and interstitial fluid mediating solute clearance in the brain, named the glymphatic system, has emerged as an important clearance pathway for neurotoxic peptides such as amyloid-beta. However, the foundational science of the glymphatic system is based on rodent studies. Here we investigated whether the glymphatic system exists in a large mammal with a highly gyrified brain. CSF penetration into the brain via perivascular pathways, a hallmark of glymphatic function, was seen throughout the gyrencephalic cortex and subcortical structures, validating the conservation of the glymphatic system in a large mammal. Macroscopic CSF tracer distribution followed the sulci and fissures showing that these folds enhance CSF dispersion. Three-dimensional renditions from light sheet microscopy showed a PVS influx density 4-fold larger in the pig brain than in mice. This demonstrates the existence of an advanced solute transport system in the gyrencephalic brain that could be utilised therapeutically for enhancing waste clearance.

## Introduction

Apart from providing cushioning and buoyancy for the brain, cerebrospinal fluid (CSF) has also been implicated in the maintenance of neural homeostasis and removal of harmful metabolites with several experiments demonstrating communication between the CSF and brain neuropil.^1–3^ The glymphatic hypothesis explains the process of advective CSF-ISF exchange for waste removal from the neuropil to the subarachnoid space (SAS).^4–6^ The glymphatic system is a brain-wide influx and clearance system formed by a network of perivascular spaces (PVS) that permit the exchange of CSF.^4^,^5^,^7^ The advective flow of CSF in the PVS and through the brain parenchyma acts to clear metabolic waste.^4^,^5^,^8^,^9^ This process is dependent on aquaporin-4 (AQP4) water channels which are highly expressed on the astrocyte endfeet that define the outer border of the PVS.^4^,^10^ The precise mechanisms of glymphatic flux and clearance are as of now incomplete but it is known that glymphatic function predominates during sleep, with influx temporally correlated with the systolic phase of the cardiac cycle, and a decline in function with age and loss of AQP4.^10–12^ However, the molecular details and knowledge of glymphatic physiology and metabolite clearance stems primarily from rodent studies, and yet rodents differ substantially from humans in neuroarchitecture and sleeping behaviour.^13–15^ The mouse brain weighs around 0.5 g and is lissencephalic while a human brain approximately 3000 times larger and is gyrified.^16^,^17^ Mice are nocturnal and sleep for multiple short periods throughout the day while most humans require an average of 8 hours of single extended bouts of sleep to maintain cognitive health.^18^,^19^ Unfortunately, glymphatic studies with the resolution to capture PVS as highways for CSF distribution are highly invasive, severely limiting the potential expansion of knowledge in human subjects. This emphasizes the need for the use of an intermediate species, easily accessible for study and more closely related to humans in order to understand the finer details of glymphatic physiology in large mammals. Interestingly, the pig brain is both closer in size and more similar in macroscopic structure to a human brain than that of a rhesus macaque.^20^ Pigs also appear to be among the mammals with a sleeping pattern that most closely resembles that of humans.^21^ While humans sleep approximately 33.3% of 24 hours, pigs are documented to sleep for approximately 32.6% of this period, with the rhesus macaque coming in at 49.2% and mice on average sleeping for 44% of this period, but highly fragmented.^21^ The pig is therefore a suitable intermediate species between mice and humans for the field of glymphatics and pushes the frontiers of our understanding of the glymphatic system and translating this understanding to humans.

To this end, we carried out CSF tracer studies in Landrace pigs, which are comparable in size to humans and have highly gyrified brains of similar macroscopic architecture. Using confocal, electron microscopy (EM) and optical clearing followed by light sheet microscopy, our experiments confirmed an extensive perivascular solute transport far exceeding the levels in mice, and thus is consistent with the existence of a highly developed glymphatic system in gyrified brains of large mammals.

## Materials and methods

### Animals

Adult male pigs, *Sus scrofa domesticus,* weighing 50–55 kg, and adult male C57BL/6 mice were used for the experiments. Mice were housed in standard laboratory conditions with a 12 h light-dark cycle, *ad libitum* access to water. Pigs were housed in two’s in pens with a 12 h light-dark cycle, *ad libitum* access to water. All experimental procedures were performed according to ethical approval by the Malmö-Lund ethical Committee on Animal Research (Dnr 5.2.18-10992/18 and Dnr 5.8.18-08269/2019) and conducted according to the CODEX guidelines by the Swedish Research Council, Directive 2010/63/EU of the European Parliament on the protection of animals used for scientific purposes and Regulation (EU) 2019/1010 on the alignment of reporting obligations. This study complies with the ARRIVE (Animal Research: Reporting in Vivo Experiments) guidelines for reporting of animal experiments.

### Anaesthesia

Pigs were first tranquilised/premedicated with an intramuscular injection of Zoletil (tiletamine 3.75 mg/kg + zolazepam 3.75 mg/kg) and Dexdomitor (dexmedetomidine 37.5 µg/kg). Once unconscious, animals were intubated and a 20 G cannula was inserted into the ear vein. For maintenance anaesthesia, a triple-drip (100 ml ketamine 100 mg/ml (5 mg/kg/min), 200 ml fentanyl 50 µg/ml (2.5 µg/kg/min), 100 ml midazolam 5 mg/ml (0.25 mg/kg/min) was applied through the ear vein until effect (± 0.5 ml/10 kg/min). Breathing was maintained at 14 breaths per minute using a Servo ventilator. All animals underwent constant monitoring for heart rate, blood pressure, oxygen saturation, pO_2_, pCO_2_ and subsequent anaesthesia infusion rate adjustment if needed. Mice received a single intraperitoneal injection of ketamine (100 mg/kg) and xylazine (20 mg/kg).

### Exposure of cisterna magna

Briefly, the skin overlying the back of the head and neck was resected. The underlying muscle layers were severed at their respective origins and retracted. Any excess tissue overlying the skull base and atlas was removed.

### Intracisternal tracer infusion

For pigs while the head of the animal was flexed, an 18 G cannula was introduced approximately 5 mm into the cisterna magna and fixed in place with glue and dental cement. 500 µL of either 1% or 2% AlexaFluor647-conjugated bovine serum albumin (BSA-647, Invitrogen) was injected using a 1 ml syringe connected to a 10 cm I.V line at a rate of 100 µL per minute. After injection, BSA-647 was allowed to circulate for 2, 4 or 6 hours. For mice, cisterna magna (CM) injection was carried out with a 30 G dental needle (Carpule, Sopira) connected to a 100 µL Hamilton syringe via PE10 tubing. 10 µL of 2% AlexaFluor647-conjugated bovine serum albumin (BSA-647, Invitrogen) tracer were injected into the CM at 1 µL/min using an KDS Legato 100 single infusion syringe pump. After injection, BSA-647 was allowed to circulate for 30 minutes.

### Tissue processing

Whole pig brains were carefully extracted by removing the dorsal skull surface with a hand-held rotating saw blade (Dremel, DSM/20) and severing the spinal cord, pituitary gland and cranial nerves with a surgical spatula. Whole brains were post fixed in 4% paraformaldehyde (PFA) for 24 hours. Brains were then sliced coronally using a salmon knife and slices were fixed in PFA for a further 24 h. For immunohistochemistry and microscopic investigations parts of the brain were sliced with a vibratome (Leica VT1200S) at a thickness of either 100 µm for hippocampus and striatum or 200 µm for the cortex.

### Optical tissue clearing

The iDISCO+ protocol was carried out as explained by Renier *et al* (2016). Pig brain pieces and whole mouse brains were dehydrated in increasing methanol/H_2_O series (20%, 40%, 60%, 80%, 100%, 100%, 1 hour each), delipidated with methanol/dichloromethane (33%/66% for 3 hours) and pure dichloromethane (2 x 15 min), and optically cleared by impregnation dibenzyl ether (DBE) for at least 14 days prior to imaging.

### Immunohistochemistry

Free-floating brain sections were permeabilized and blocked for 45 min at 4 °C in a solution of 1% BSA, 0.5% Triton X-100 and 5% normal donkey serum in PBS. Primary antibodies (rabbit anti-AQP4, 1:500, MerckMillipore; rabbit anti-GFAP, 1:500, Agilent Technologies; mouse anti-GLUT1, 1:250, Abcam, rabbit anti-SMA, 1:500, AbCam) were added in PBS and incubated overnight at 4 °C on a rocking table. After 3x10 min washes in PBS at room temperature secondary antibodies (Alexa-Fluor 488- and 568-conjugated secondary antibodies, 1:1000) in PBS were added for 90 minutes at 4 °C on a rocking table. Slices were then washed in PBS with DAPI (1:1000) and/or tomato lectin (Lycoperiscon esculentum, 1:20, SigmaAldrich) for 20 minutes, washed again and mounted.

### Imaging

Whole brains and macroscopic slices were imaged using a Nikon SMZ25 stereomicroscope with a Plan Apo 0.5x objective (0.08 NA) equipped with an Andor Zyla 4.2 Plus sCMOS camera (Mag-0.75x, Zoom-1.5x). The excitation wavelength was 635 nm using a CoolLED pE4000 LED illumination and the emission filter used was a quadruple bandpass filter.

Vibratome slices were imaged with both Nikon Ti2 Eclipse and Nikon A1RHD confocal microscopes. Cleared pig brain tissue and whole cleared mouse brains were imaged using an Ultramicroscope II light-sheet microscope (LaVision Biotech) with a 1.3X LaVision LVMI-Fluor lens (0.105 NA) equipped with an sCMOS camera (Andor Neo, model 5.5-CL3). The excitation wavelength was 640 nm and the emission filter used was 680/30 nm. Brain pieces were imaged immersed in DBE in the transverse orientation at a z-step size of 5 µm with ImspectorPro64 (LaVision Biotec).

### Analyses

For all paired measurements, the mean intensity of a specific region (sulcus, fissure, brain region) was compared to the mean intensity of the whole area of interest (dorsal brain surface, lateral brain surface, whole slice) in the same brain. For independent comparisons, ratios of a specific region of interest (sulcus, fissure, brain region) divided by the mean intensity of the whole area of interest (dorsal brain surface, lateral brain surface, whole slice) in the same brain were used. Pig 3 was excluded from sulcul surface analyses owing to a surface congenital malformation. For “midline shift”, a line was drawn from and perpendicular to the interhemispheric fissure in the posterior cerebral cortex out the lateral brain border; the intensity around the IHS fissure was used as a maximum value and the distance in % from the IHS fissure to the lateral border for the tracer intensity to reach 50% of the maximum value was used; an average was generated between both cortices in a single brain. Data was normalised within each sample for whole brain and microscopic slice analyses to account for differing imaging parameters based on differing circulation times. For vessel peak plots, a line was drawn through the vessel in an image stack containing each staining; a plot was generated for each fluorophore and all plots were then overlaid on a single graph. For PVS counting, orthogonal views to the inner cortical surface were generated to gain a cross-sectional view of the spaces. Briefly, background was reduced, signal threshold applied, then converted to binary using a mask and particles counted to generate PVS number per mm^2^ and % area coverage. All images were analysed using Fiji.^22^

### Immunoelectron microscopy

Pig cortical tissues (1 mm x 2 mm) were pre-fixed in a solution containing 1% PFA and 1.5% glutaraldehyde in 0.1 M phosphate buffered saline for 3 h at room temperature and then rinsed several times prior to fixation with 1% osmium tetroxide. This was followed by dehydration with acetone (30–100%), impregnation and embedding in pure Epon for sectioning (60 nm). Immunostaining was performed using primary antibody against BSA (1:1000, Sigma Aldrich) and secondary antibody conjugated to 10 nm gold nanospheres (1:20, Abcam). Sections were stained with 4% uranyl acetate for 20 mins followed by 0.5% lead citrate for 2 minutes to increase the contrast of the tissue. Sections were examined using FEI Tecnai Biotwin 120kv transmission electron microscope (TEM) and photographed using Olympus Veleta 2x2k camera at magnifications ranging from 2000–30,000×.

### Statistics

All statistics were performed on GraphPad Prism 8 (GraphPad Software). Data were tested for normality using Shapiro-Wilk test. Two-tailed paired t-tests or two-tailed students t-tests were used for comparing two groups. Multiple groups were analysed using a repeated measures one-way ANOVA with Tukey’s multiple comparison post hoc test. All values are expressed as mean/mean difference ± SD. N represents number of biological replicates. P < 0.05 was accepted as statistically significant.

## Results

### Sulci facilitate extensive CSF distribution

**Figure 1.**
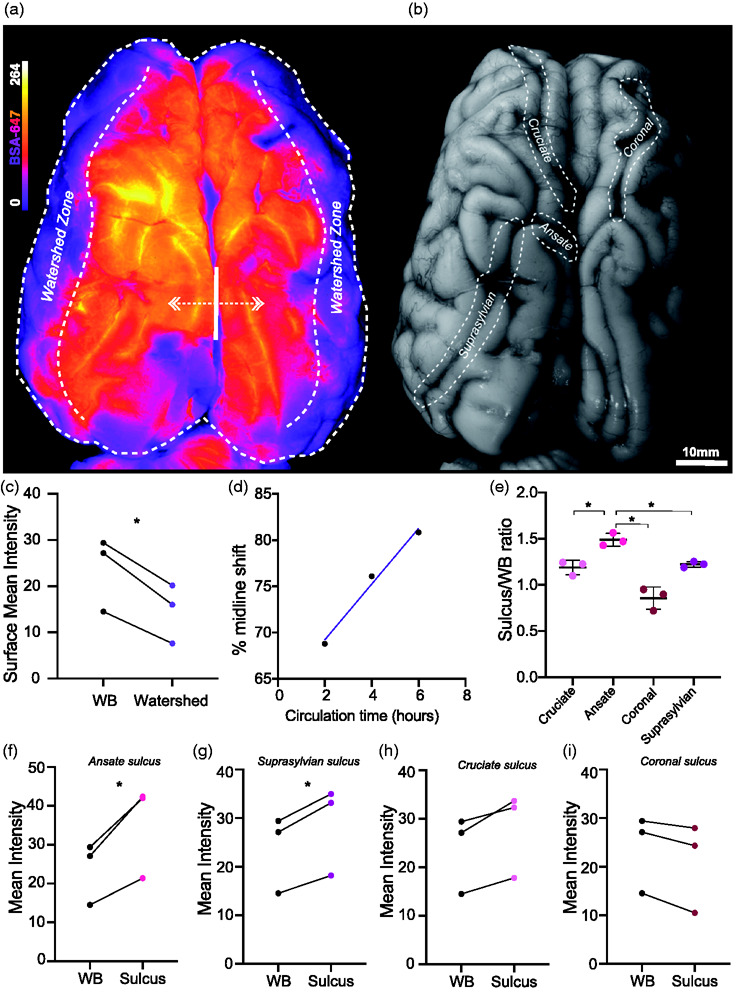
CSF tracer distribution at the dorsal brain surface. (a) Representative image of whole dorsal surface (WB) of pig brain under fluorescent light showing BSA-647 tracer distribution. (b) Representative image of whole dorsal surface of pig brain under white light. (c) Surface BSA-647 tracer intensity over whole dorsal surface versus watershed zone (two-tailed paired *t*-test, p = 0.0178). (d) “Midline shift” vs circulation time. Percentage distance from IHS fissure to lateral aspect of brain where tracer reaches 50% value of IHS value. (e) Comparison of sulcus/whole brain intensity ratio amongst cruciate, ansate, coronal and suprasylvian sulci (repeated measures one-way ANOVA with Tukey’s multiple comparison). (f) Surface BSA-647 tracer intensity over whole dorsal surface versus ansate sulcus (two-tailed paired *t*-test, p = 0.0433). (g) Surface BSA-647 tracer intensity over whole dorsal surface versus suprasylvian sulcus (two-tailed paired *t*-test, p = 0.0199). (h) Surface BSA-647 tracer intensity over whole dorsal surface versus cruciate sulcus (two-tailed paired *t*-test, p = 0.0674). (i) Surface BSA-647 tracer intensity over whole dorsal surface versus coronal sulcus (two-tailed paired *t*-test, p = 0.0671). N = 3. *p<0.05. Graphs represent mean ± SD. BSA-647, Alexa Fluor 647 conjugated to bovine serum albumin. WB, Whole Brain.

Injection of tracers in the cisterna magna (CM) permits them direct access to the SAS, which contains CSF and covers the entire surface of the brain. We investigated the macroscopic profile of CSF distribution and whether there were any patterns or favoured paths. The entire cerebral hemisphere of a mammalian brain is supplied by the posterior (PCA), middle (MCA) and anterior (ACA) cerebral arteries and their branches.^23^ Areas where end branches meet and anastomose are called watershed zones and are typically more vulnerable to cerebral ischaemia.^24–26^ We compared mean tracer intensity in the watershed zone, where the branches of the middle and anterior cerebral artery meet to the whole dorsal surface and found that tracer values in this zone were consistently 50% lower, even in the brains with longer circulation times (mean diff. = 9.097 ± 2.133, p = 0.0178, two-tailed paired *t*-test; Figure 1(a) and (c)). This observation made it apparent that the most distal arterial branches might not exhibit the same capacity for perivascular distribution as the major cerebral arteries and their initial branches. Thus, these areas may not only be more vulnerable to ischaemia but could also have a greater propensity for waste aggregation in instances of reduced glymphatic function. Influx differences in the watershed zone did not differ significantly with circulation time so we further addressed this parameter by taking the tracer intensity around the interhemispheric fissure as a maximum intensity value and determined how far laterally tracer travelled before amounting to 50% of the maximal value, here termed “midline shift.” Midline shift yielded a trend with longer circulation times amounting to a greater shift laterally in tracer intensity (Figure 1(a) and (d)). Thus, with longer circulation times, tracer tends to be transported further laterally from the midline but not all the way to the watershed zone, further reaffirming the role of large calibre arteries and their early branches in driving CSF distribution.^12^,^27^ Light sheet imaging of a cleared volume of pig cortex additionally highlighted large vessels as a key vehicle in transmitting tracer influx deeper into the brain cortex (Supplementary video 1).

An evolutionary advancement in the brains of higher mammals is the development of sulci and gyri from the folding of the brain.^28^ Sulci increase the tissues’ surface area, which our data shows could also impact CSF distribution. On the dorsal surface of the pig brain there are at least 4 major sulci: cruciate, ansate, coronal and suprasylvian.^29^ We determined whether any of the major pig sulci were preferential paths for CSF by comparing the mean tracer intensity surrounding each sulcus at the surface level (Figure 1(b)). When comparing amongst the sulci, the tracer intensities were significantly higher in the ansate sulcus compared to the cruciate (p = 0.0199), coronal (p = 0.0391) and suprasylvian (p = 0.0477) sulci (repeated measures one way-ANOVA; Figure 1(e)). When comparing sulci intensities to the entire dorsal surface, both the ansate (mean diff. = 11.6 ± 4.422, p = 0.0433, two-tailed paired *t*-test; Figure 1(f)) and the suprasylvian (mean diff. = 5.097 ± 1.265, p = 0.0199, two-tailed paired *t*-test; Figure 1(g)) sulcus exhibited significantly higher tracer intensities. At the surface level, the cruciate sulcus showed a tendency for higher tracer intensity values (mean diff. = 4.262 ± 2.020, p = 0.0674, two-tailed paired *t*-test; Figure 1(h)) while, interestingly, tracer intensity values tended to be lower in the coronal sulcus when compared to the entire dorsal cerebrum (mean diff. = -2.758 ± 1.304, p = 0.0671, two-tailed paired *t*-test; Figure 1(i)). These intriguing findings indicate that brain gyrification is favourable for extensive CSF dispersion throughout the cortical surface.

### Widespread cortical penetration of CSF tracer

**Figure 2.**
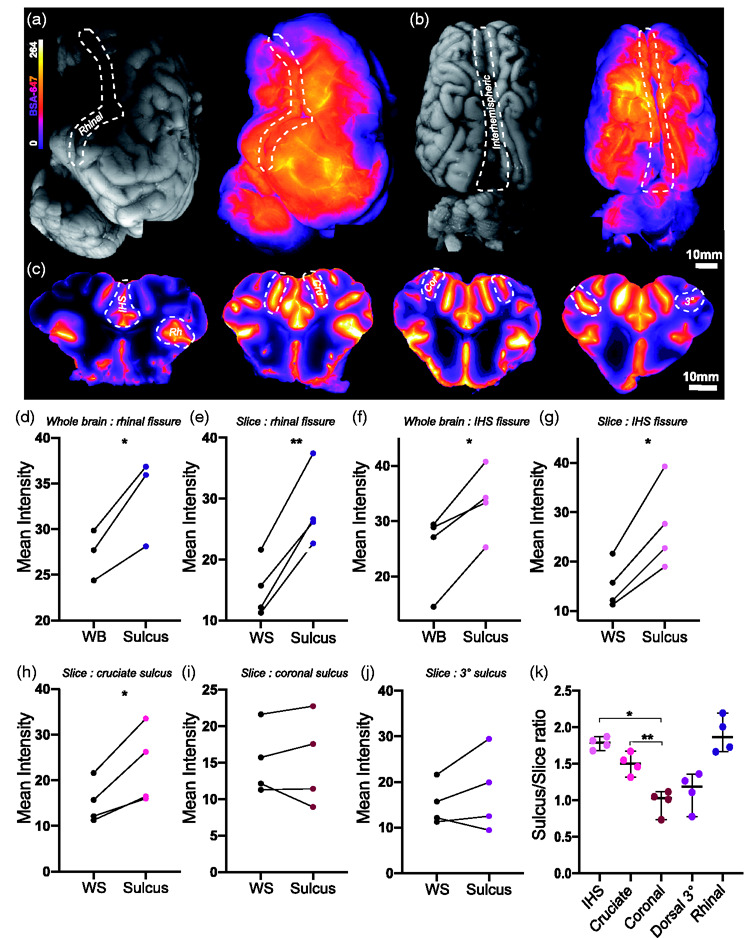
Macroscopic imaging of slices give greater insights into CSF tracer distribution. (a) Representative images of whole lateral surface of pig brain under white light and under fluorescent light showing BSA-647 tracer distribution. (b) Representative images of whole dorsal surface of pig brain under white light and under fluorescent light showing tracer distribution. (c) Representative images of macroscopic brain slices showing tracer distribution. (d) Surface tracer intensity over whole lateral surface versus rhinal fissure. (two-tailed paired *t*-test, p=0.0422, N=3). (e) Tracer intensity over whole slice versus rhinal fissure. (two-tailed paired *t*-test, p=0.002). (f) Surface tracer intensity over whole dorsal surface versus interhemispheric fissure (two-tailed paired *t*-test, p=0.0137). (g) Tracer intensity over whole slice versus interhemispheric fissure. (two-tailed paired *t*-test, p=0.0107). (h) Tracer intensity over whole slice versus cruciate sulcus (two-tailed paired *t*-test, p=0.0281). (i) Tracer intensity over whole slice versus coronal sulcus (two-tailed paired *t*-test, p=0.9794). (j) Tracer intensity over whole slice versus 3° sulcus (two-tailed paired *t*-test, p=0.3219). (k) Comparison of sulcus/whole slice intensity ratio amongst interhemispheric fissure, cruciate sulcus, coronal, 3° sulcus and rhinal fissure (repeated measures one-way ANOVA with Tukey’s multiple comparison). N=4. *p<0.05, **p<0.01, ***p<0.001, ****p<0.0001. Graphs represent mean ± SD. BSA-647, Alexa Fluor 647 conjugated to bovine serum albumin; Cor, Coronal; Cru, Cruciate; HIS, Interhemispheric; Rh, Rhinal; WB, Whole brain; WS, Whole slice; 3°, Tertiary.

To achieve imaging of the full extent of the sulci and their capacity for CSF distribution, we examined two of the large fissures, interhemispheric and rhinal, both at the surface level and from a macroscopic slice level (Figure 2(a) to (c)). The macroscopic slices demonstrated the extent of tracer penetration into both the fissures and sulci (Figure 2(c)). Compared to the whole lateral surface of the brain (WB), the rhinal fissure yielded significantly higher tracer intensities (mean diff. =6.322 ± 2.323, p = 0.0422, two-tailed paired *t*-test; Figure 2(d)). This observation was further reinforced at the slice level comparing the fissure intensity to the whole slice (WS), with a mean difference between groups at the slice level more than double that of the surface level (mean diff. = 13.03 ± 2.538, p = 0.0002, two-tailed paired *t*-test Figure 2(e)). The interhemispheric fissure yielded similar results at both surface (mean diff. = 8.415 ± 3.229, p = 0.0137, two-tailed paired *t*-test; Figure 2(f)) and slice levels (mean diff. = 11.96 ± 4.195, p = 0.0107, two-tailed paired *t*-test; Figure 2(g)). This shows that at the given circulation times, more CSF moves through the SAS overlying these regions than other surrounding regions. Similarly in humans, brain regions adjacent to the IHS fissure and Sylvian/lateral fissure, comparable to the rhinal fissure in pigs, appear to exhibit earlier and more intense tracer enrichment than surrounding regions.^27^ This could be due to lower fluid resistance derived from more potential space within sulci in combination with arterial pulsations from branches of the large calibre arteries that run within fissures and sulci.

The same analyses were carried out on 3 other sulci at the slice level: the cruciate, coronal and so-called “3°” sulcus. Of these only the cruciate sulcus exhibited significantly higher values than the whole slice (mean diff. = 7.881 ± 3.945, p = 0.0281, two-tailed paired *t*-test; Figure 2(h) to (j)). Comparing tracer intensities between the sulci and fissures revealed that fissures (IHS and rhinal) tended to receive more CSF tracer than the sulci (repeated measures one-way ANOVA; Figure 2(k)).

**Figure 3.**
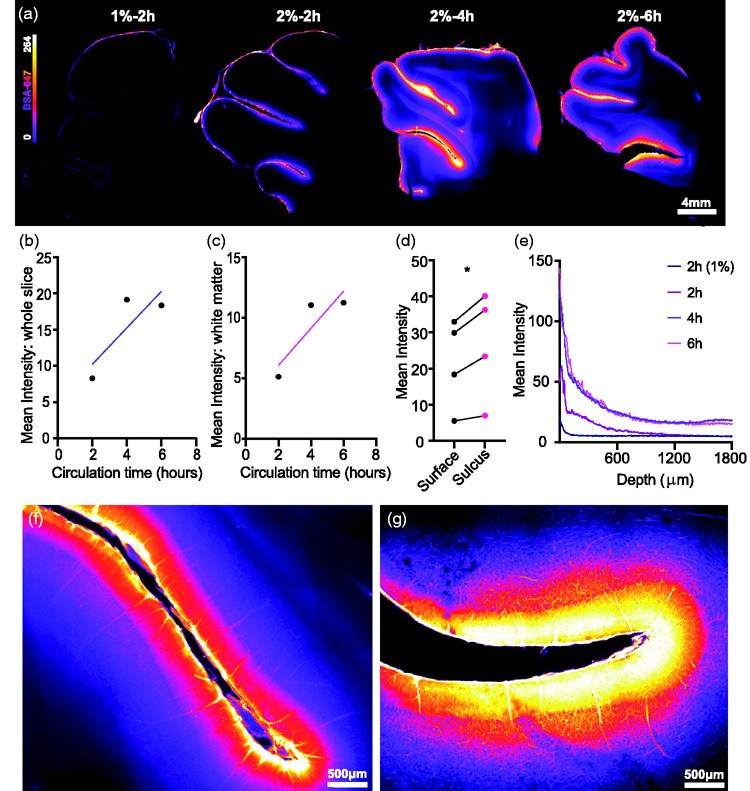
Glymphatic influx predominates at sulci. (a) Representative images of vibratome brain slices demonstrating glymphatic influx. (b) Slice intensity vs circulation time with trend for increased intensity based on circulation. (c) White matter slice intensity vs circulation time with trend for increased intensity based on circulation. (d) BSA-647 tracer intensity over exposed surface vs tracer intensity over of sulcul surface (two-tailed paired *t*-test, p=0.0283, N=4). (e) Mean pixel intensity from slice surface to 1800 µm into the slice across circulation times and BSA-647 tracer intensities. (f) Representative 20X magnified epiflourescent image of slice sulcus. (g) Representative 20X magnification image of slice sulcus using confocal. *p<0.05. BSA-647, Alexa Fluor 647 conjugated to bovine serum albumin.

Next, tissue bounding the interhemispheric fissure was sectioned and imaged for insights into glymphatic penetration (Figure 3(a)). Tracer circulation time showed a trend for increased overall slice intensity (Figure 3(b)) and white matter fluorescence intensity (Figure 3(c)). At this microscopic level we also compared tracer intensities along the slice surface to tracer intensities within the sulci, with values in the sulci consistently higher (mean diff. = 4.995 ± 2.508, p = 0.0283, two-tailed paired *t*-test; Figure 3(d)). This phenomenon was further explored through optically clearing a piece of pig cortex and light sheet imaging and the preference of tracer toward and within a sulcus as compared to the surface was evident (Supplementary video 2). Finally, we addressed depth of tracer penetration, a proxy for glymphatic influx, versus tracer concentration and tracer circulation time and observed that longer circulation times yielded trends for both higher tracer surface intensities along with deeper penetration (influx) and more gradual reductions in tracer intensity at regions more distal from the surface (Figure 3(e)). High magnification epifluorescence and confocal microscopy images further exhibited a dense perivascular network of glymphatic influx in the large mammalian brain (Figure 3(f) to (g)).

The increased tracer intensity in sulci compared to the surface and gyri was clearly visible across imaging modalities which validates the role of these structures in directing CSF, highlighting how the anatomical differences in the gyrencephalic brain as compared to the lissencephalic brain can functionally influence CSF paths.

### CSF penetrates the porcine cortex via perivascular routes

**Figure 4.**
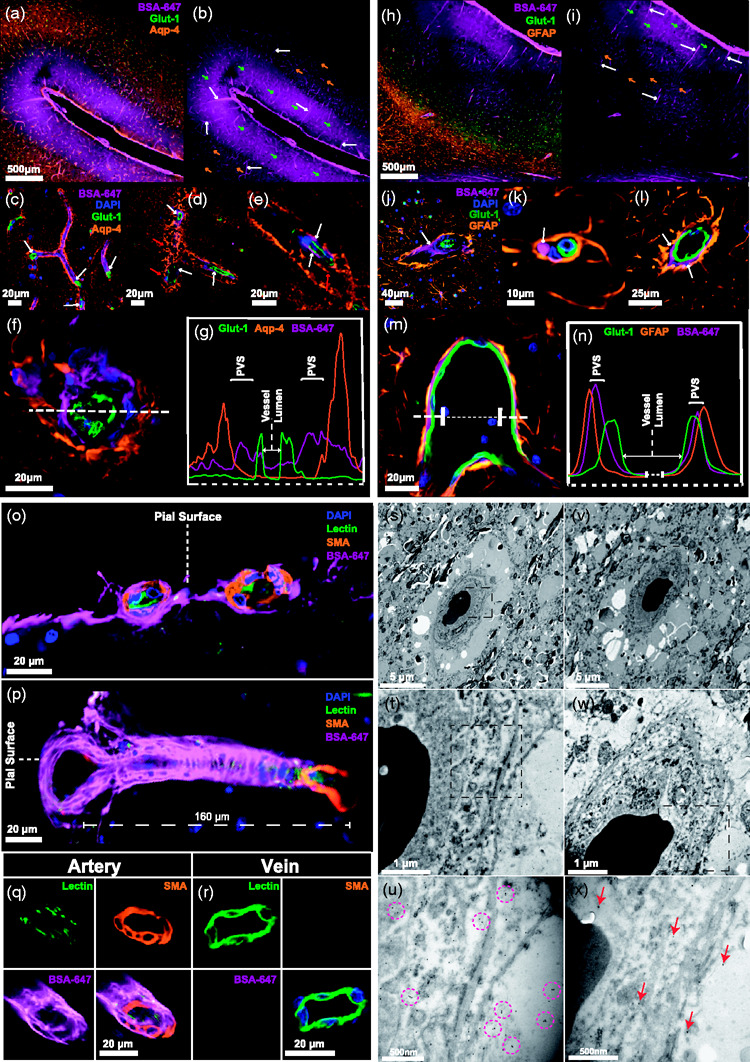
CSF penetrates the porcine cortex via perivascular routes. (a) Representative image of immunohistochemical staining with Glut-1 for endothelial cells and AQP4 with tracer (BSA-647). (b) Representative image of CSF tracer distribution. White arrows point to tracer in perivascular space (PVS). Green arrows show higher amounts of tracer present in the parenchyma near the surface. Orange arrows show lower amounts of tracer visible deeper in the parenchyma. (c-e) 20x magnified images of tracer in the PVS along vessels. White arrows point to perivascular tracer. Red arrows point to AQP4 architecture. (f) Cross section through cortical vessel showing perivascular tracer ensheathed by AQP4. (g) Intensity profiles of AQP4, BSA-647 and Glut-1 across the vessel cross-section. (h) Representative image of immunohistochemical staining with Glut-1 (blood vessels) and GFAP (astrocytes) with tracer (BSA-647). (i) Representative image of tracer distribution. White arrows show tracer in perivascular space (PVS). Green arrows point to higher amounts of tracer present in the parenchyma near the surface. Orange arrows point to lower amounts of tracer visible deeper in the parenchyma. (j-l) 20x magnification images showing tracer in the PVS along vessels. White arrows point to perivascular tracer. (m) Cross section through cortical vessel showing perivascular tracer ensheathed by GFAP. (n) Intensity profiles of GFAP, BSA-647 and Glut-1 across the vessel cross-section. (o) Cross section through two surface pial arteries stained with smooth muscle actin (SMA) showing CSF-injected BSA-647 surrounding the vessels. (p) Cross section through sulcul pial artery stained with SMA showing pathway for BSA-647 entry into the parenchyma along penetrating artery branch. (q-r) Cross section through artery and vein approximately 900 µm from the brain surface with artery identified through SMA staining and presence of BSA-647. (s-u) Electron microscopy images of a single vessel in pig cortex stained with primary antibodies against tracer and secondary antibodies coupled to gold nanoparticles. Purple circles highlight specific clusters of immunogold staining. (v-x) Electron microscopy images of a single vessel in pig cortex stained with only secondary antibodies coupled to gold nanoparticles as control. Red arrows point to single gold particles. BSA-647, Alexa Fluor 647 conjugated to bovine serum albumin; D, Dorsal; DG, Dentate gyrus; EHC, Entorhinal cortex; Hp, Hippocampus; SMA, Smooth muscle actin; Str, Striatum; V, Ventral.

PVS drastically increase the surface area through which the CSF communicates with the brain and are believed to efficiently facilitate the movement of CSF throughout the neuropil and clearance of waste products.^4^,^12^ The inner bounds of the PVS are formed by endothelial cells while astrocytic endfeet giving rise to the glia-limitans form the outer bound. To visualise tracer in the perivascular spaces of the pig brain, we stained for GLUT-1 (endothelial cells) and AQP4 (Figure 4(a)). Tracer was evident both along vessels and in the surrounding parenchyma as a haze in upper cortical layers. More distal from the cortical surface, the amount of tracer in the parenchyma lessened while perivascular tracer remained unequivocally present (Figure 4(b)). Higher magnification confocal images of endothelial cells surrounded on the outer rim by dense AQP4 staining exhibited clearly visible tracer running along the longitudinal aspects of vessels (Figure 4(c) to (e)). A complex three-dimensional AQP4 architecture contributing to a bulky perivascular space was seen surrounding vessels of approximately 20 µm in diameter (Figure 4(d) to (f)). By plotting an intensity profile for each fluorophore, three distinct peaks demonstrated that CSF tracer is bounded by an endothelial cell and AQP4 peak either side, constituting the PVS (Figure 4(g)). Similar results emerged when using a GFAP staining for astrocytes (Figure 4(h) to (n)) and thus these findings demonstrate the presence of the integral microscopic machinery of the glymphatic system as described in rodents now identified in a large mammal. To classify the blood vessels implicated in the glymphatic pathway, further stainings were carried out against smooth muscle actin (SMA), which binds to smooth muscle cells in arterial walls (Figure 4(o) to (r), Supplementary Figure 1). This helped identify pial arteries at the brain surface and their penetrating branches as initial sites for CSF distribution (Figure 4(o) and (p)). Deeper into the neuropil tracer was identified surrounding smaller arterioles approximately 20  μ m in diameter but not around veins of a similar size (Figure 4(q) to (r)). This reaffirms that CSF influx takes place in the periarterial space in rodents as well as large mammals.

To obtain even higher resolution images, we used immunogold labelling against the CSF tracer (bovine serum albumin) and performed electron microscopy (EM). Imaging at 2,000–30,000X on a transmission EM (TEM) platform allowed for the identification of blood vessels and the brain tissue surrounding them (Figure 4(s) to (x)). In order to control for non-specific retention of gold nanoparticles we carried out staining with (Figure 4(s) to (u)) and without (Figure 4(v) and (x)) primary antibodies. While non-specific retention of gold particles in control samples yielded randomly dispersed singular particles (Figure 4(x)), specific immunogold staining was visible in the experimental group identifiable as several clusters of 2–3 gold nanoparticles in the anatomical region circumventing the vessel (Figure 4(u)). This further strengthens our findings that tracer moves into the brain along blood vessels and this phenomenon persists down to a capillary level with immunogold staining.

### Subcortical favouritism of hippocampal CSF influx

**Figure 5.**
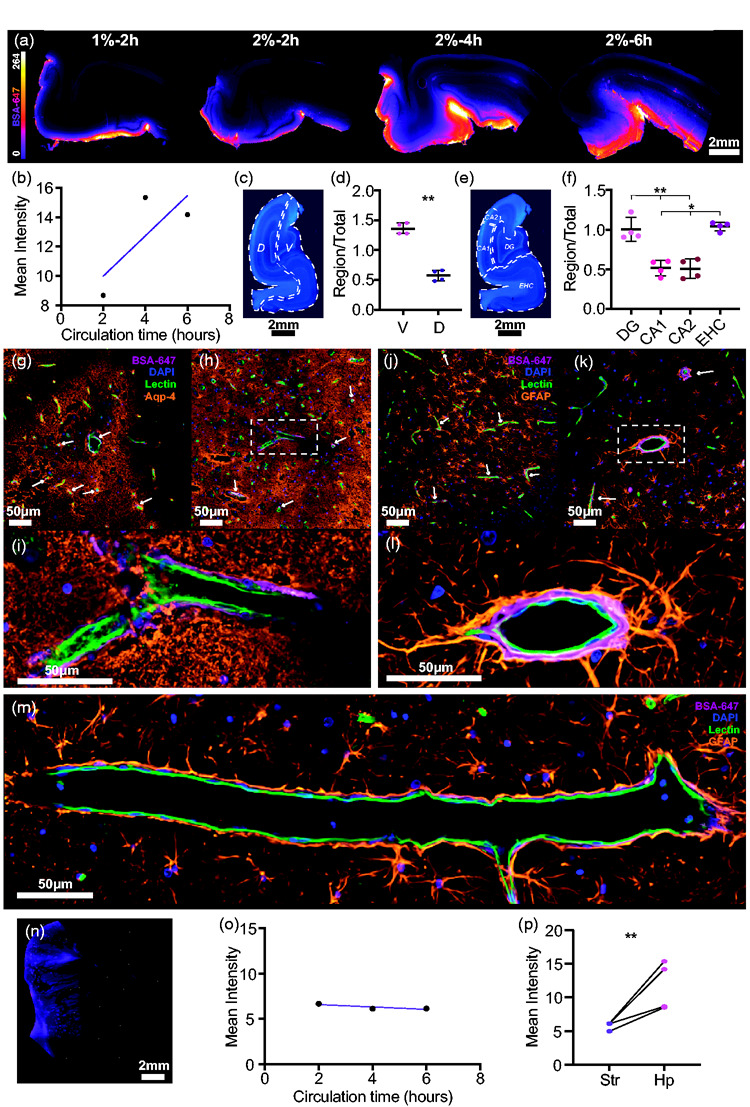
Glymphatic influx reaches hippocampus before striatum. (a) Representative images of vibratome hippocampal slices showing glymphatic influx. (b) Overall hippocampal intensity vs circulation time. (c) Representative image of pig hippocampus stained with DAPI showing dorsal/ventral division. (d) Comparison of regional vs whole slice intensity ratios for dorsal and ventral hippocampal regions (two-tailed paired *t*-test, p = 0.0034). (e) Representative image of pig hippocampus stained with DAPI showing regional divisions. (f) Comparison of regional vs whole slice intensity ratios for CA1, CA2, entorhinal cortex and dentated gyrus (repeated measures one-way ANOVA with Tukey’s multiple comparison). (g-i) Representative images of hippocampus stained with AQP4, lectin and DAPI with BSA-647 tracer. White arrows point to tracer in PVS. (j-l) Representative images of hippocampus stained with GFAP, lectin and DAPI with tracer. White arrows point to tracer in PVS. m 20x magnified image of hippocampus showing longitudinal-sectional face of blood vessel stained with lectin with tracer in the PVS and GFAP staining for astrocytes. (n) Representative image of pig striatum. (o) Overall striatal intensity vs circulation time. (p) Comparison of mean striatal vs mean hippocampal intensity (two-tailed paired *t*-test, p = 0.0376). N = 4. **p<0.01, ***p<0.001. Graphs represent mean ± SD. BSA-647, Alexa Fluor 647 conjugated to bovine serum albumin.

To investigate subcortical glymphatic influx in the pig, we dissected and sliced the hippocampus from each brain (Figure 5(a)). Longer incubation times yielded a trend for increased tracer influx in the hippocampus. Figure 5(b)). In order to understand how glymphatic function is favoured in the hippocampus it was divided into ventral and dorsal regions (Figure 5(b) and (c)). The ventral aspect of the hippocampus exhibited significantly more glymphatic influx than the dorsal region (mean diff. = 0.786 ± 0.1839, p = 0.0034, two-tailed paired *t*-test; Figure 5(d)). Both the dentate gyrus (DG) and the entorhinal cortex (ERC) exhibited significantly more tracer influx than in CA1 and CA2, which were both comparable (repeated measures one-way ANOVA; Figure 5(e) and (f)). These findings can be resolved in an anatomical sense as the ventral aspect of the hippocampus, and thus the ventral regions like the DG and ERC, are in direct communication with the ambient cistern. Thus, CSF and tracer have more direct and immediate access to the hippocampus.

Vascular staining in combination with AQP4 or GFAP showed the network of astrocytes in the hippocampus. GFAP immunoreactivity revealed that astrocyte endfeet visibly projected to the PVS and CSF tracer was visible within the AQP4 lining surrounding hippocampal vessels (Figure 5(g) to (m)).

Interestingly, in contrast to glymphatic influx in the hippocampus, which showed a trend to increase with longer circulation times, influx into the striatum did not differ between 2, 4 or 6 hours (Figure 5(n) and (o)). Furthermore, overall CSF tracer influx was significantly higher in the hippocampus compared to the striatum (mean diff. = 5.842 ± 3.274, p = 0.0376, two-tailed paired *t*-test; Figure 5(p)). This is in keeping with a previous study in humans, where hippocampal tracer enrichment began to increase after 2 hours finally peaking around 8 hours and tracer enrichment in the basal ganglia peaked at only 48 hours.^27^

### Perivascular influx density in pigs is more extensive than in rodents

**Figure 6.**
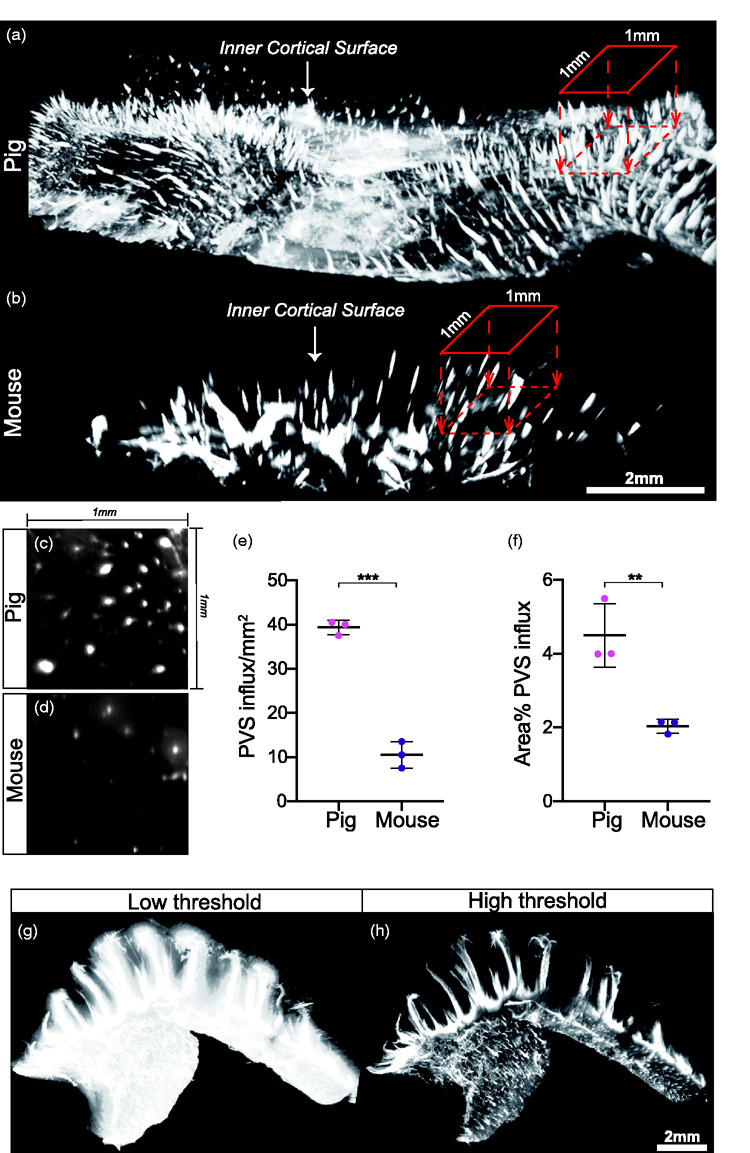
The extent of CSF tracer distribution in the pig PVS network exceeds that in mice. (a-b) 3D reconstruction of pig and mouse inner cortical surface highlighting perivascular influx sites. 1mm by 1mm region of interest is used to count perivascular influx sites projecting into the brain. Scale bar = 2mm. (c-d) Representative image of a cross section through perivascular channels parallel to cortical surface in pig brain and mouse brain. (e-f) Quantification of number of perivascular influx sites per mm^2^ of cortical surface in pig and mouse brains (students *t*-test, p<0.0001) and percent of cortical surface area covered by perivascular influx in pig and mouse brains (students *t*-test, p=0.0086). (g-h) 3D renditions of pig hippocampus from light sheet microscope at low and high thresholds. N=3. **p<0.01, ***p<0.0001. Graphs represent mean ± SD.

Light sheet microscopy of optically cleared tissues enables imaging of whole tissue volumes at optical resolution without the need for tissue sectioning. Recently, our lab showed that visualisation of the glymphatic system in entire mouse brains is possible using optical tissue clearing and light sheet microscopy and provides a full overview of glymphatic architecture.^7^ To compare the glymphatic system in pigs and mice in undisturbed brain tissue, we applied optical clearing and light sheet microscopy which revealed a pattern of regularly distributed entry points of CSF tracer from the cortical surface into the brain (Figure 6(a) and (b)). Quantification of the density of PVS in the cortical surface was 4-fold greater in pigs compared to mice (39.33 ± 1.607 vs 10.5 ± 3 PVS/mm^2^, p < 0.0001, students *t*-test; Figure 6(c) to (e)). This further translated to a superior PVS area-coverage of cortical surface (4.497 ± 0.8648 vs 2.037 ± 0.1897% area coverage, p = 0.0086, students *t*-test; Figure 6(f)). The vast extent of CSF distribution to perivascular spaces in the pig brain was further visible in three dimensional renderings of the cortical surface (Supplementary videos 3 and 4).

To explore CSF tracer influx in a region with a specialised tissue architecture, hippocampi were optically cleared and light sheet imaged. A low threshold reconstruction exhibited pervasive CSF tracer influx into the entire hippocampal volume while a high threshold visualised the primary pathways which produced this pervasive influx (Figure 6(g) and (h)). These primary pathways for CSF influx can be further appreciated in 3 D renderings of the entire sub-cortical volume (Supplementary videos 5 and 6).

In summary, optical clearing and light sheet microscopy revealed a highly organised pattern of CSF entry into the brain across species and that the perivascular space CSF transport network has a higher density in the gyrencephalic brain.

## Discussion

The recently discovered glymphatic system is a brain clearance pathway that has been implicated in the removal of toxic peptides such as Aβ. Perivascular spaces represent a key constituent of the glymphatic system, providing an extensive network for CSF-ISF exchange.^4^,^5^ The vast majority of all investigations into the glymphatic system and our most fundamental knowledge of it stems from rodent studies.^4^,^10^,^11^,^30–34^ The few studies carried out in humans utilized MRI and were valuable in demonstrating macroscopic cortical influx patterns, yet lacked the spatial resolution to demonstrate the microscopic glymphatic machinery such as perivascular influx and polarised expression of AQP4.^27^,^35–37^ Similar macroscopic contrast enhancement was also revealed in macaques after CM injection with gadolinium chelate.^38^ Herein, we demonstrated the extent of the glymphatic system in a gyrified brain from a large mammal, ranging from specific patterns of CSF paths at the whole brain level, thought to be mediated by a folded architecture in combination with arterial pulsations from large calibre blood vessels, down to the perivascular localisation of CM-injected tracers in both the cortex and subcortical structures. Furthermore, we have used light sheet imaging of cleared brain volumes to visualise the three-dimensional glymphatic architecture of the gyrencephalic brain at optical resolution which enabled a direct comparison of the extent of the PVS network across species.

Interestingly, glymphatic influx of tracer was significantly reduced in the MCA-ACA watershed zone in the large gyrencephalic brain. These border zones represent the most terminal areas of the cortical vascular fields and are the first to develop infarcts upon generalised cerebral hypoperfusion.^39^ Our data further demonstrates that as well as being susceptible to hypoperfusion these zones may also yield a less pronounced glymphatic influx and subsequent clearance. Cerebral hypoperfusion is a well-established consequence of AD and more recently a significant association was found between watershed cortical infarcts and AD.^26^,^40^ Thus, these cumulative effects of infarction coupled to reduced glymphatic function could lead to more pronounced degeneration of neural tissue in the watershed zone and aggregate the overall progression of AD.

Apart from brain size a notable difference between rodents and pigs is the absence of sulci and gyri in the rodent brain. Our data suggests that the sulci play an important role in CSF distribution with evident fluorescent tracer dispersion in these folds. However, tracer intensities were not equal across all sulci. The folds proximal to the IHS and lateral fissure housed more tracer, and thus transmit more CSF. Thus, it appears that the fissures act as primary sites for CSF distribution, akin to national roadways, wherefrom CSF then flows through the most proximal connected sulci, which act as offramps, and so on downstream to further connected sulci. We maintain that this pattern of CSF distribution occurs because of the size of the potential space and the proximity to a major cerebral artery. Both fissures represent the largest potential spaces across the cortical surface and thus offer least resistance to the flow of fluid i.e CSF. Additionally, each fissure houses the ACA and MCA, respectively, and their most proximal branches, which have been shown to be strong propagators of CSF.^12^,^27^ In the gyrencephalic brain penetrating arteries run from pial arteries at the cortical surface and from within the sulci, and so the CSF transmitted via the sulci has rapid access to a vast network of penetrating influx sites. In this way the sulci not only increase the macroscopic distribution of CSF but also increase the amount of surface area for glymphatic influx.

Similar to the differential CSF distribution seen over the cortex so too were differences observed in the hippocampus, with significantly higher tracer intensities present across the ventral aspect of the hippocampus. The reason for this robust influx may be due to the ventral aspect of the hippocampus being in direct communication with the ambient cistern. From this cistern CSF is able to directly penetrate this aspect of the hippocampus via ventral penetrating arteries. Additionally, the PCA runs inferiorly to the ventral aspect of the hippocampus and sends up branches which give rise to longitudinal arteries running along the ventral aspect of the hippocampus, whose pulsations assist to further propagate tracer to ventral penetrating arteries.^41^ Several transverse arteries arise from the longitudinal artery and travel to dorsal aspect of the hippocampus.^42^ The increased glymphatic influx at the ventral aspect of the hippocampus may in fact be an evolutionary protective measure based on the selective vulnerability of the DG and other ventral limbic regions like the subiculum and EHC, which appear to be more susceptible to severe Aβ deposition.^43^

The definition of the PVS is commonly brought into question and to what extent it extends down penetrating arteries. Regardless of the definition what is certain is that CSF does penetrate the neuropil in an area encircling these arteries, perivascular or other. Through the use of immunogold staining we were able to address the extent to which CSF surrounding arteries/arterioles penetrates the brain and found nanoparticles encircling microvasculature approximately 5 μ m in diameter. This qualitatively suggests that the extent of CSF propagation along vessels progresses substantially deeper than the penetrating arteries.

Finally, not only have we demonstrated the existence of microscopic glymphatic pathways in pigs but also performed light sheet microscopy of optically cleared pig brain tissue. Along with increased sulci-based CSF distribution, the 4-fold increase in PVS influx density in the pig brain suggests a significantly greater propensity for convective CSF transport, and downstream of this, glymphatic function. Human studies using intrathecal gadobutrol have invaluably demonstrated macroscopic centripetal patterns of cortical tracer enrichment over relatively short time frames of 24–48 h.^27^,^35^ Additionally it has been shown that intrathecal injections of gadobutrol exhibit a degree of contrast enhancement exceeding that predicted by diffusion modelling.^37^ Our data potentially explains how such a rapid tracer enrichment is possible in a large gyrencephalic brain, attributable both to the attenuated sulci-based CSF distribution coupled to the 4-fold increase in the density of cortical PVS. These architectural phenomena may account for the rapid contrast enhancement seen in human studies and shows that the route from CSF spaces to the brain parenchyma occurs via PVS. Thus, the impairment of brain tracer enrichment and clearance in idiopathic normal pressure hydrocephalus could be contributed to by microscopic pathology impacting PVS influx density.^27^,^44^ Additionally, infarcted tissue in stroke patients may also lead to reduced PVS influx density which could impair glymphatic clearance in surrounding tissue and worsen stroke outcome.^45^

## Limitations

A critical difference when comparing the PVS density between the mouse and pig was the different anaesthetic regimes used for either surgery. Since the pig is a large mammal it requires a more advanced regime which included ketamine, an opiate and a benzodiazepine, administered continuously through a central line, while the mouse simply received a single intraperitoneal injection of a ketamine-xylazine (KX) cocktail due to the lethal respiratory inhibitory effects of opiates on such a small mammal. However, this should not detract from the data showing a 4-fold increase in PVS influx density in the pig based on the fact that of all anaesthetic regimes tested the KX cocktail has robustly been shown to be the most effective agent for facilitating glymphatic function and CSF movement through the PVS.^46^ Thus, KX being the gold standard regime for glymphatics supports that whatever has been shown in the pig, based on rodent studies, could likely be an underestimation of maximal glymphatic function. A supplementary concern when introducing exogenous tracers into the CSF is raised intracranial pressure (ICP).We acknowledge that the intrathecal administration of tracers into the CSF could raise the ICP, however at low volumes this elevation is momentary with normalisation to baseline in minutes.^47^,^48^ Furthermore, if the injection is given at low volumes over time as opposed to a bolus dose ICP is not significantly elevated.^47^ In mice, it is acceptable to inject 10 µL with an infusion rate of 1 µL/min for glymphatic studies.^7^,^10^ Translating this to the pigs, whose brain mass is approximately 100 g, it would be comparable to inject tracer at a rate of 200 µL/min amounting a final volume of 2 mL. Instead, we injected ¼ of the translated acceptable volume at ½ the infusion rate and it is thus unlikely that maintained pathological elevations in ICP occurred and that our observations reflect results from aberrations in ICP.

## Conclusions

Expanding links continue to be made in rodents between ageing and impaired glymphatic function and this fits with the hypothesis that lack of glymphatic function contributes to neurodegeneration.^11^,^49^ This is encouraging in terms of potentially utilizing glymphatic function as a therapeutic target against neurodegeneration, however, the only supportive evidence collected from humans is a post mortem study which revealed that AQP4 polarisation had a strong association with AD status and pathology.^50^ As such the potential role of glymphatic function in neurodegeneration would benefit from being further explored in large mammals with a brain similar to that of humans. Through this research we have taken a stride forward in uncovering the microscopic glymphatic pathways in the gyrencephalic brain which may closely resemble the glymphatic pathways that exist in the human brain, and thereby shed light on the more likely architecture of human glymphatics. In this way, our work serves as a base to progress in validating findings from rodents and in doing so maximise the possibility of an eventual effective translation in glymphatic therapeutics to humans.

## Supplemental Material

sj-pdf-1-jcb-10.1177_0271678X21996175 - Supplemental material for Glymphatic pathways in the gyrencephalic brain Click here for additional data file. Supplemental material, sj-pdf-1-jcb-10.1177_0271678X21996175 for Glymphatic pathways in the gyrencephalic brain by Nicholas Burdon Bèchet, Nagesh C Shanbhag and Iben Lundgaard in Journal of Cerebral Blood Flow & Metabolism

sj-pdf-2-jcb-10.1177_0271678X21996175 - Supplemental material for Glymphatic pathways in the gyrencephalic brain Click here for additional data file. Supplemental material, sj-pdf-2-jcb-10.1177_0271678X21996175 for Glymphatic pathways in the gyrencephalic brain by Nicholas Burdon Bèchet, Nagesh C Shanbhag and Iben Lundgaard in Journal of Cerebral Blood Flow & Metabolism
