# Supplementary figures and images for: Glymphatic pathways in the gyrencephalic brain
Source: J Cereb Blood Flow Metab. 2021 Feb 27;41(9):2264–79. doi: 10.1177/0271678X21996175 (PMC8393296; doi:10.1177/0271678X21996175)

# Cortex

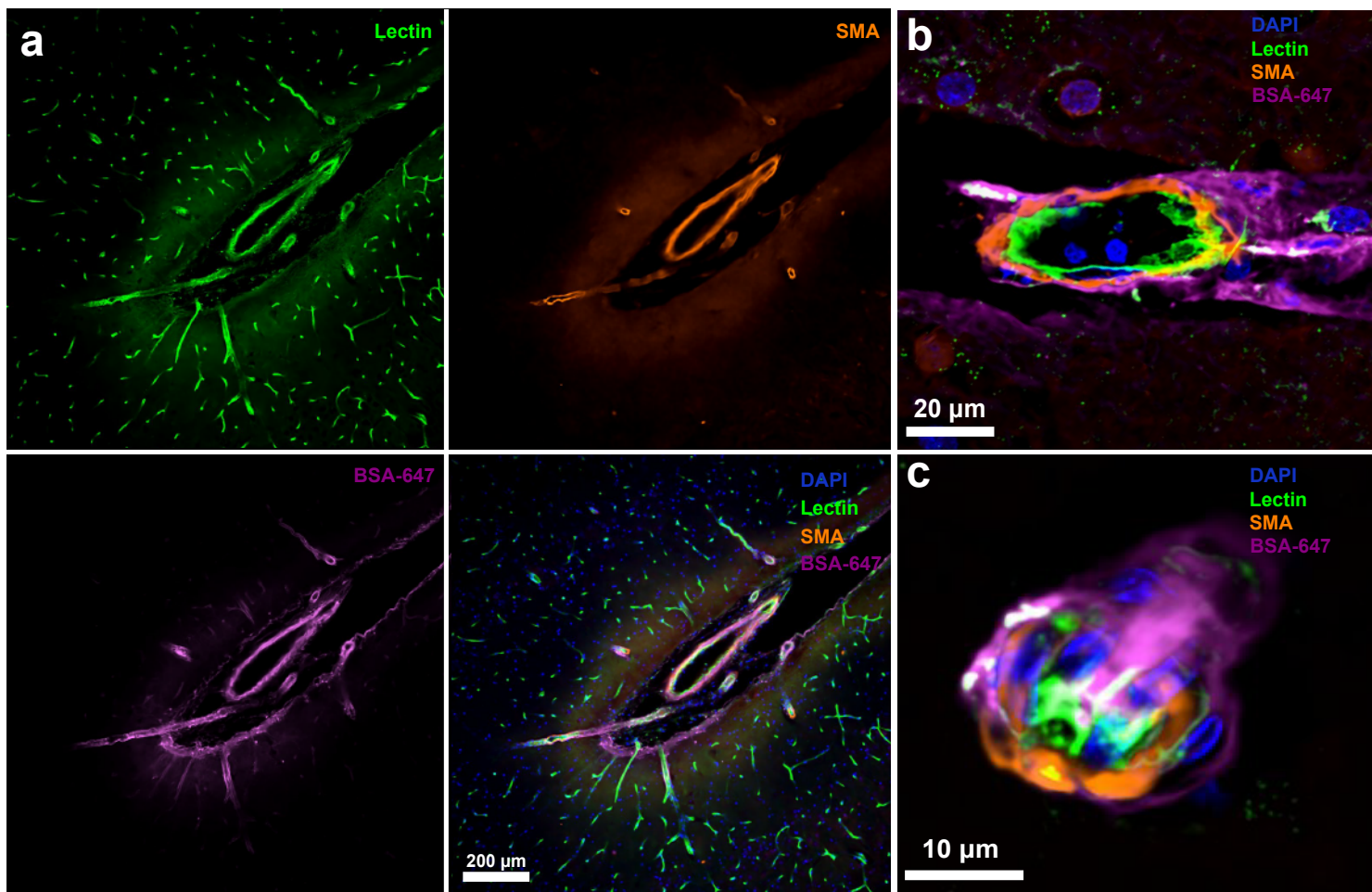

# Hippocampus

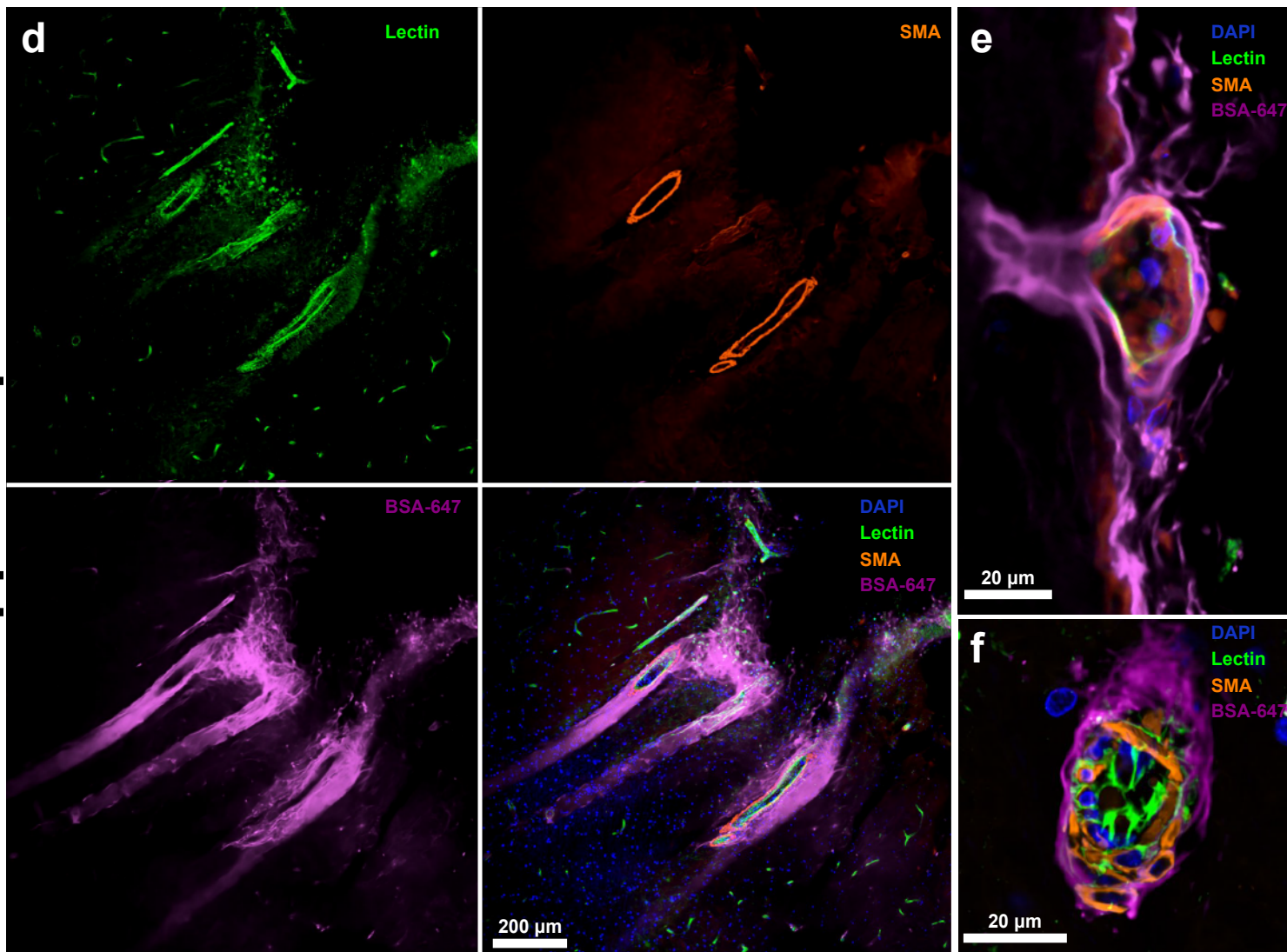

Supplement: sj-pdf-1-jcb-10.1177_0271678X21996175 - Supplemental material for Glymphatic pathways in the gyrencephalic brain [file sj-pdf-1-jcb-10.1177_0271678X21996175.pdf]
