# Supplementary material for: Glymphatic pathways in the gyrencephalic brain
Source: J Cereb Blood Flow Metab. 2021 Feb 27;41(9):2264–79. doi: 10.1177/0271678X21996175 (PMC8393296; doi:10.1177/0271678X21996175)
Supplement: sj-pdf-2-jcb-10.1177_0271678X21996175 - Supplemental material for Glymphatic pathways in the gyrencephalic brain [file sj-pdf-2-jcb-10.1177_0271678X21996175.pdf]

**SI APPENDIX**

**Supplementary Figure 1. Periarterial tracer influx in cortex and hippocampus**

(**a**) Periarterial tracer influx from large pial arteries at sulcul surface and along penetrating branches into pig brain cortex. (**b-c**) Periarterial tracer influx in cortical arterioles in pig 3 and pig 4. (**d**) Periarterial tracer influx from large penetrating arteries at hippocampal ventral surface. (**e**) Pial artery at hippocampal ventral surface with surrounding tracer. (**f**) Periarterial tracer influx in hippocampal arteriole. BSA-647, Alexa Fluor 647 conjugated to bovine serum albumin; SMA, Smooth muscle actin.

**Supplementary video 1:** Light sheet reconstruction of pig cortex exhibiting tracer influx along the PVS of a large calibre vessel.

**Supplementary video 2:** Light sheet reconstruction of pig cortex exhibiting more intense tracer distribution in sulcus than cortical surface.

**Supplementary video 3:** Light sheet reconstruction of inner pig cortex demonstrating extensive influx of tracer into brain via regularly distributed perivascular channels.

**Supplementary video 4:** Light sheet reconstruction of inner mouse cortex demonstrating influx of tracer into brain via regularly distributed perivascular channels but less than in pig.

**Supplementary videos 5-6:** Light sheet reconstruction of whole pig hippocampi exhibiting primary tracer influx paths from ventral aspect and along large calibre vessels.
